# Receptor-Based Pharmacophore Modelling of a series of ligands used as inhibitors of the SARS-CoV-2 virus by complementary theoretical approaches, molecular docking, and reactivity descriptors.

**DOI:** 10.12688/f1000research.133426.1

**Published:** 2023-06-26

**Authors:** Alejandro Morales-Bayuelo, Jesús Sánchez-Márquez

**Affiliations:** 1 Grupo GENOMA, Escuela de Medicina, Universidad del Sinú-EBZ, Cartagena, Colombia; 2 Departamento de Química-Física, Universidad de Cadiz, Cádiz, Andalusia, Spain

**Keywords:** RNA dependent RNA polymerase SARS-CoV-2 virus, COVID-19 treatments, molecular docking, chemical reactivity descriptors, density functional theory.

## Abstract

**Background:** A *coronavirus* identified in 2019, *SARS*- *CoV*- *2*, has *caused* a pandemic of respiratory *illness*, called *COVID*- *19.* Most people with COVID-19 experience mild to moderate symptoms and recover without the need for special treatments. The SARS‑CoV‑2 RNA‑dependent RNA polymerase (RdRp) plays a crucial role in the viral life cycle. The active site of the RdRp is a very accessible region, so targeting this region to study the inhibition of viral replication may be an effective therapeutic approach. For this reason, this study has selected and analysed a series of ligands used as SARS-CoV-2 virus inhibitors, namely: the Zidovudine, Tromantadine, Pyramidine, Oseltamivir, Hydroxychoroquine, Cobicistat, Doravirine (Pifeltro), Dolutegravir, Boceprevir, Indinavir, Truvada, Trizivir, Trifluridine, Sofosbuvir and Zalcitabine.

**Methods:** These ligands were analyzed using molecular docking, Receptor-Based Pharmacophore Modelling. On the other hand, these outcomes were supported with chemical reactivity indices defined within a conceptual density functional theory framework.

**Results:** The results show the conformations with the highest root-mean-square deviation (RMSD), have π-π stacking interaction with residue LEU141, GLN189, GLU166 and GLY143, HIE41, among others. Also was development an electrostatic potential comparison using the global and local reactivity indices.

**Conclusions:** These studies allow the identification of the main stabilizing interactions using the crystal structure of SARS‑CoV‑2 RNA‑dependent RNA polymerase. In this order of ideas, this study provides new insights into these ligands that can be used in the design of new COVID-19 treatments. The studies allowed us to find an explanation supported in the Density Functional Theory about the chemical reactivity and the stabilization in the active site of the ligands.

## Introduction

COVID-19 is an infectious disease caused by SARS-CoV-2 virus. The majority of individuals infected with COVID-19 experience moderate to mild symptoms and can recover without requiring any special treatment. Nonetheless, there are some cases where people develop severe symptoms and may require medical attention. ^1^

There is still no curative treatment for COVID-19, but we already have vaccines that, in many cases, prevent infection, and in the event of contracting the disease, allow to pass through it mildly. Other treatments are still in the development phase and are intended to prevent transmission.

Treatment for severely ill COVID-19 patients and those at risk of severe disease involves administering oxygen. ^2(a)^ Critically ill patients receive more advanced respiratory support, such as mechanical ventilation. Dexamethasone, a corticosteroid, can assist in reducing the time patients spend on a ventilator and saving the lives of those with severe or critical conditions. For further information, refer to the question-and-answer section on dexamethasone. ^2(b)^ It has been demonstrated that hydroxychloroquine does not provide any therapeutic benefit against COVID-19. For more information, see the questions and answers section on hydroxychloroquine.

The self-administration of antibiotics or any other medication is not recommended by the World Health Organization (WHO) to prevent or cure COVID-19. ^3^ For these reasonsnew alternatives for COVID-19 treatment is needed. In this work, zidovudine, tromantadine, pyramidine, oseltamivir, hydroxychoroquine, cobicistat, doravirine (Pifeltro), dolutegravir, boceprevir, indinavir and zalcitabine were assessed. Some of antecedents related to ligands are: Zidovudine ^4^ is a medication used in combination with other drugs to manage human immunodeficiency virus (HIV) infection. Tromantadine ^5^ is a medication used to treat herpes simplex virus. It inhibits both early and late events in the virus replication cycle and is considered an antiviral medicine. Pyrimidine depletion and the immune response are associated with human coronavirus infection. ^6^ Oseltamivir ^7^ is used to treat some types of influenza in individuals who have experienced flu-like symptoms for no more than two days. Hydroxychloroquine ^8^ has been evaluated in clinical trials as a treatment for COVID-19, but the results have been inconclusive and must be interpreted with caution due to limitations in study design.

Several protease inhibitors have been developed to target human immunodeficiency virus 1 (HIV-1), such as cobicistat/doravirine (Pifeltro). ^9^^,^^10^ Other drugs identified by *in silico* methods with the capacity to bind to Mpro and with potential action against infection by SARS-CoV-2 include dolutegravir and boceprevir. ^11^^,^^12^ Finally, potential antivirals against SARS-CoV-2 have been proposed using virtual screening methods, such as indinavir and zalcitabine. ^13^^,^^14^ All these ligands have been analysed using theoretical techniques such as molecular docking, electrostatic potential analysis and chemical reactivity descriptors within the Density Functional Theory (DFT). ^15^

## Methods

### System preparation

The receptor structure for the docking experiment was extricated utilizing the following protocols through the crystal Structure of SARS-CoV-2 RNA-dependent RNA polymerase PBD code: 6m71, which was adjusted utilizing the protein preparation wizard module of the Schrödinger suite 2017-1. i) The optimization of the hydrogen bond (H-bond) network and refinement of the protein structure was performed. ii) PropKa utility was utilized to determine the protonation states at physiological pH. iii) The restrained molecular minimization with heavy atoms constrained to a low root-mean-square deviation (RMSD) from the initial coordinates was carried out using the Impact Refinement (Impref) module. ^16^^–^^18^

Alternatively, the molecular structures of the compounds were sketched using Maestro Editor (Maestro, version 11.1, Schrödinger, LLC). Then 3D conformations were gained with the LigPrep module, with ionization/tautomeric states predicted under physiological pH conditions with Epik. Subsequently, energy minimization was used using the protocol with the Macro model using the OPLS2005 force field.

### Molecular docking

The docking investigations were carried out using Glide ^19^^,^^20^ with the default parameters and the Standard Precision (SP) model. The docking grid was generated with the co-crystallized ligand at the center using the default settings. To enable the binding of larger ligands, a scaling factor of 0.8 was applied to the van der Waals radii of the nonpolar protein atoms. The Extra Precision (XP) was also employed for induced fit docking (IFD) to allow the protein to undergo backbone, side-chain, or both movements upon ligand docking and to expand alternate receptor conformations suitable for binding ligands with unusual orientations. Finally, considering the extent of residue movement induced by the IFD computation, to evaluate the ligands, we examined the conformations of the most and least active compounds for each molecule in the molecular set. The best predictions of the poses were predicted by 10ns molecular dymane calculations, in order to analyse its stabilization in the active site.

### Chemical reactivity analysis

Several previous investigations have established a correlation between quantum similarity and chemical reactivity descriptors. ^21^^–^^31^ Quantum similarity and DFT utilize the density function as an object of study for similarity indexes. Specifically, the Coulomb index can be linked to electronic factors related to chemical reactivity. The global reactivity indices, such as chemical potential ( *μ*), ^32^ hardness ( *ɳ*), ^33^ and electrophilicity ( *ω*), ^34^^,^^35^ will be calculated using Frontier Molecular Orbitals (FMO) and the energy gap. These chemical reactivity indices (Equations 1- 5) provide information about the stability of the systems. Chemical potential measures the tendency of electrons to leave the equilibrium system, ^36^ while chemical hardness measures the resistance of a chemical species to change its electronic configuration. ^29^

$$\mu \approx \frac{E_{\text{LUMO}}+E_{\text{HOMO}}}{2}$$
(1)

$$\eta \approx E_{\text{LUMO}}-E_{\text{HOMO}}$$ ( **2)**

The mathematical definition of the electrophilicity index ( *ω*) is related to the stabilization energy of a system when it becomes saturated by electrons from the external environment ^34^^,^^35^:

$$\omega =\frac{\mu^{2}}{2\eta }$$
(3)

For this study, the local reactivity descriptors utilized were the Fukui functions. Equations (4, 5) depict how the chemical potential of a system responds to changes in the external field. This is defined as the derivative of the electronic density with respect to the number of electrons at a constant external field.

$$f^{+}\left(\vec{r}\right)\approx {\left|\text{LUMO}\left(\vec{r}\right)\right|}^{2}$$
(4)

$$f^{-}\left(\vec{r}\right)\approx {\left|\text{HOMO}\left(\vec{r}\right)\right|}^{2}$$
(5)

The nomenclature $f^{+}\left(\vec{r}\right)$ and $f^{-}\left(\vec{r}\right)$ have been used to indicate nucleophilic and electrophilic attacks, respectively. ^36^^–^^38^ This methodology employs global and local reactivity descriptors to analyze quantum similarity in the molecular set. All calculations were carried out using the method B3LYP ^39^ and the basis set 6-311xxG(d,p) ^40^ which is the result of adding a correction to the 6-311G(d) basis set leading to calculations of electronegativity, hardness, reactivity indices and frontier molecular orbitals and is comparable in quality to those obtained with much larger basis sets (such as Aug-cc-pVQZ and Aug-cc-pV5Z). This method/basis set has been used in combination with the Gaussian 16 package. ^41^

## Results and discussion

### Molecular analysis: pharmacophore development

**Figure 1.**
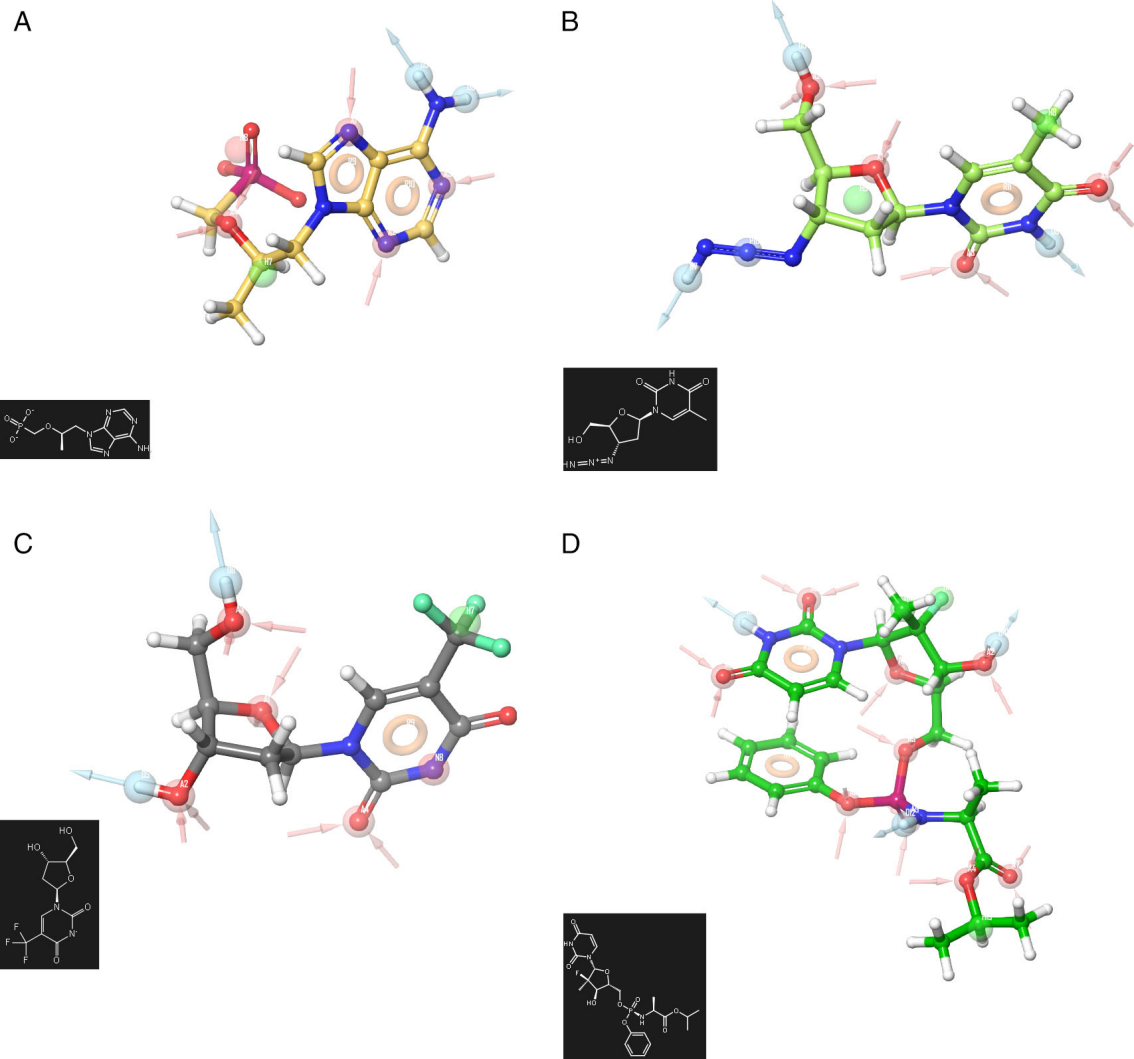
Pharmacophore development for the ligands analysed. A) Truvada, B) Trizivir, C) Trifluridine and D) Sofosbuvir.

To obtain a deeper analysis about the features associated to the biological activity the pharmacophore development has been used using the Zidovudine, Tromantadine, Pyramidine, Oseltamivir, Hydroxychoroquine, Cobicistat, Doravirine (Pifeltro), Dolutegravir, Boceprevir, Indinavir, Truvada, Trizivir, Trifluridine, Sofosbuvir and Zalcitabine. Figure 1 shows the pharmacophore development using the ligands: **A)** Truvada, **B)** Trizivir, **C)** Trifluridine and **D)** Sofosbuvir. To develop a pharmacophore model, the co-complexed ligand was extracted from its original conformation in the protein and subjected to the pharmacophore development tool available in the Schrödinger suite 2017-1. ^16^

**Figure 2.**
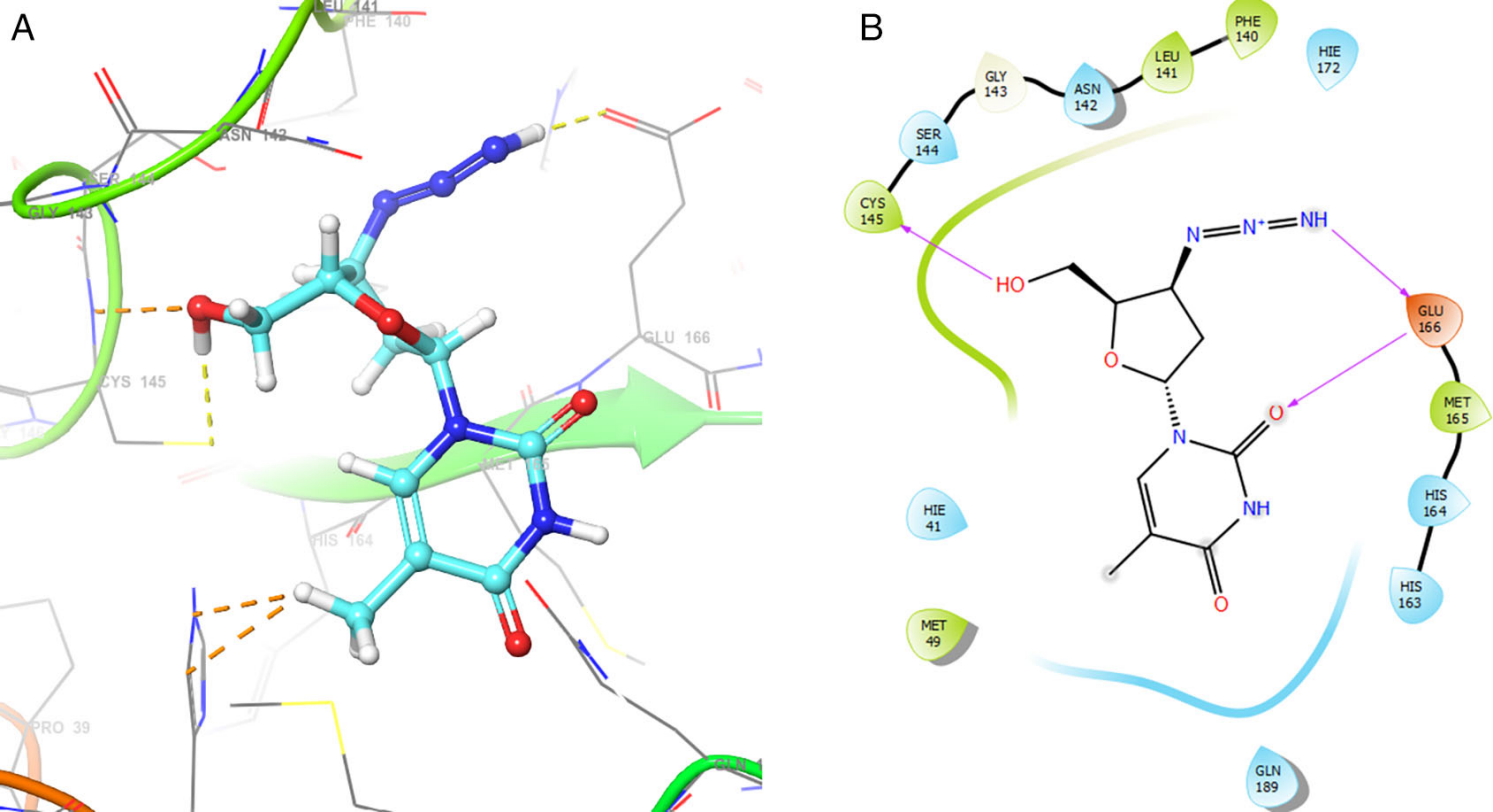
Molecular docking outcomes (A) and interaction (B) for zidovudine into de pocket protein crystal structure of SARS-CoV-2 RNA-dependent RNA polymerase PBD code: 6m71.

A receptor-based pharmacophore model was constructed ( Figure 1) to identify key features associated with biological activity, namely negative, positive, and aromatic ring. The model incorporated hydrogen bond acceptor, hydrogen bond donor, and hydrophobic features, which showed good agreement with a previously reported model. The validation process yielded an RMSD value of 0.38. Figure 2 shows the molecular docking outcomes for the ligand studied.

Figure 2 shows the H-bonds for the best conformation of zidovudine with the residues CYS145 and GLU166. For this ligand the best pose has been with docking score -6.241. The H-bond is formed with the alcohol group with the residue CYS145 and ketone group for the residue GLU166.

**Figure 3.**
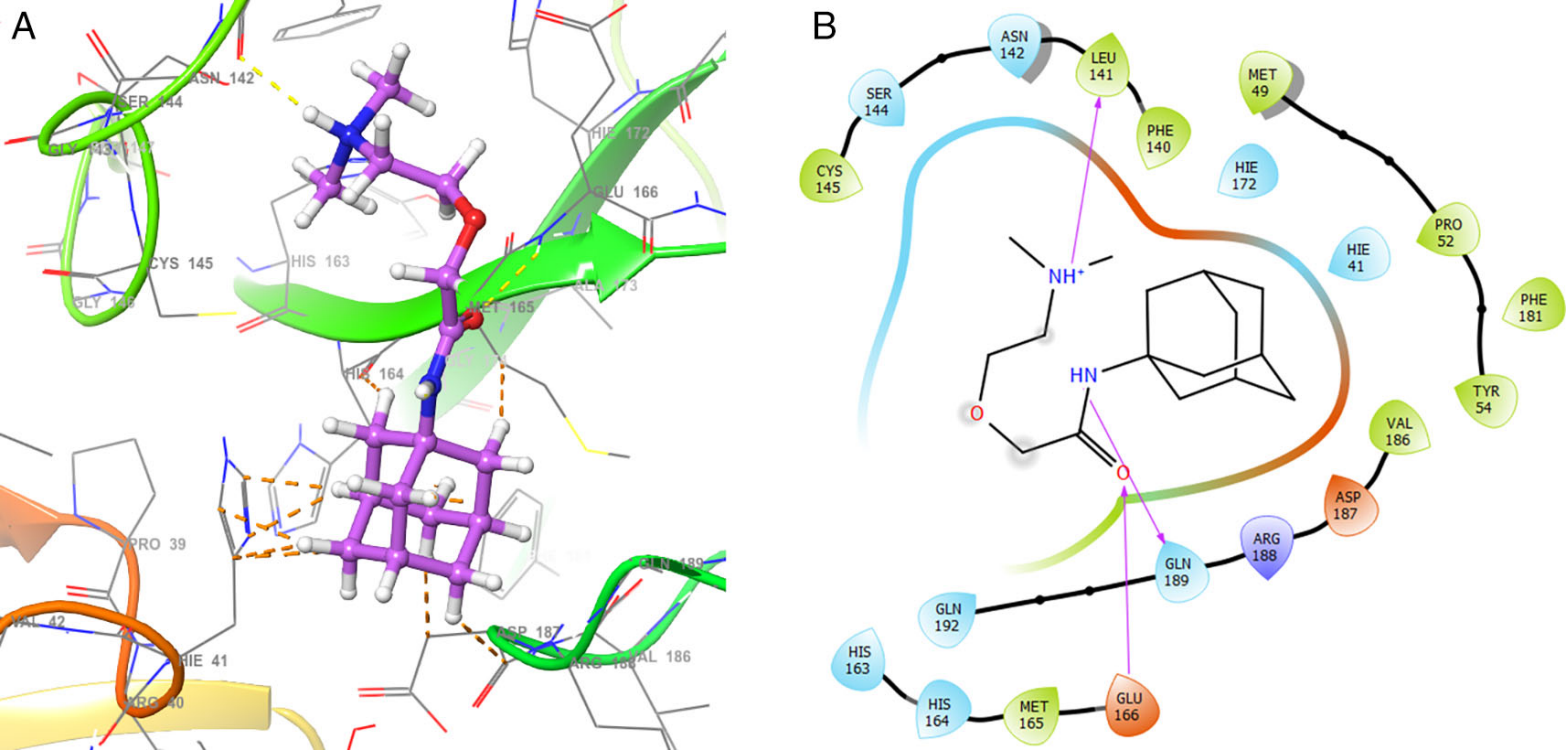
Molecular docking outcomes (A) and interaction (B) for tromantadine.

As seen in Figure 3, tromantadine showed H-bonds with the residue LEU141 with bond length 1.62Å, GLN189 with bond length 1.54Å and with the residue GLU166 with bond length 1.59Å. The H-bonds with the residues LEU141 and GLN189 are with the amino groups and ketone group for the residue GLU166, like Zidovudine.

**Figure 4.**
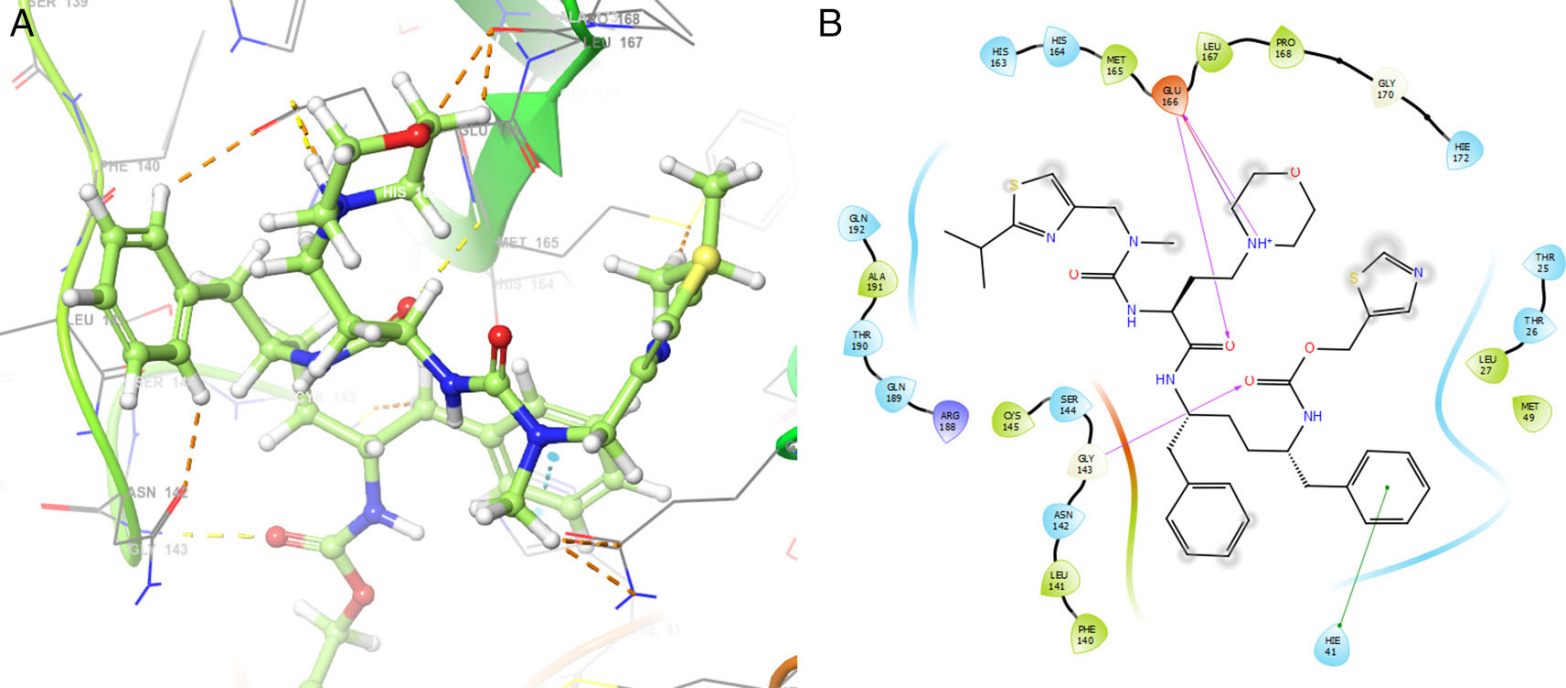
Molecular docking outcomes (A) and interaction (B) for cobicistat.

Figure 4 shows the H-Bond with the residues GLY143 with length 1.53Å, GLU166 with length 1.61Å and HIE41 with length 1.71Å. The GLY143 and GLU166 had H-bonds with the ketone group, HIE41 had H-bond with the aromatic ring, and finally GLU166 had a H-bond with an amino group.

**Figure 5.**
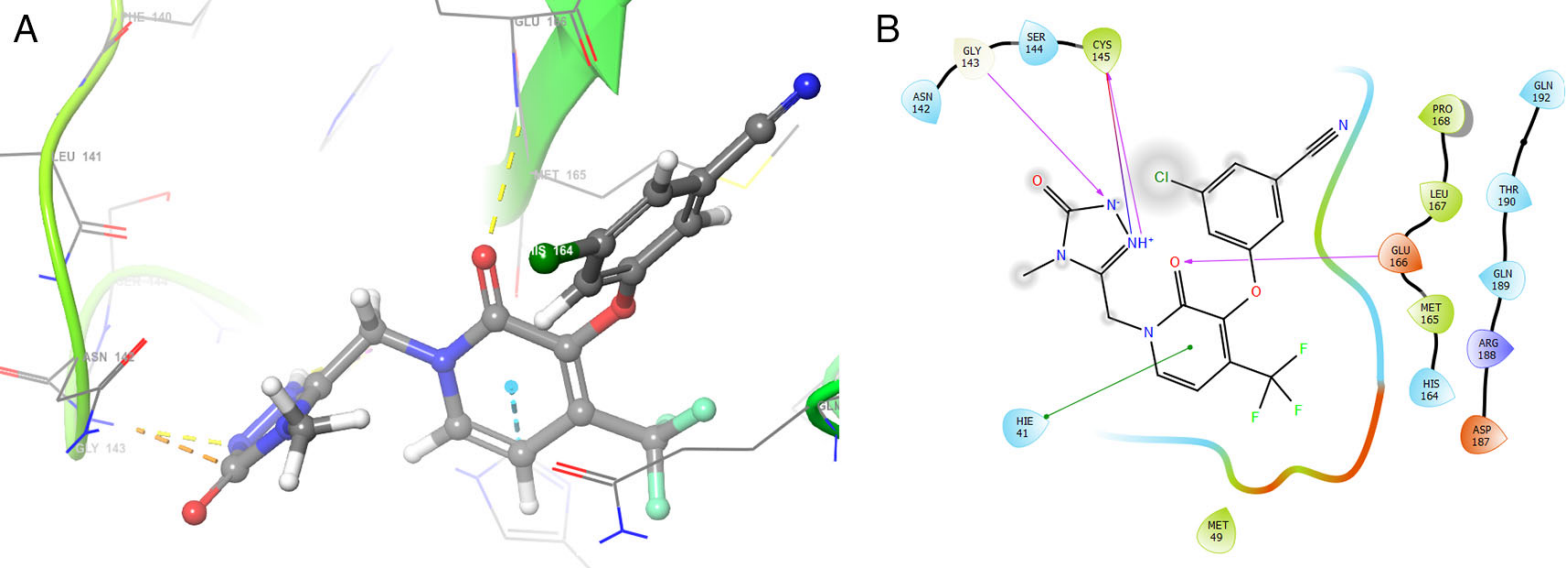
Molecular docking outcomes (A) and interaction (B) for doravirine (Pifeltro).

On the other hand, Figure 5 shows the H-bonds for doravirine. This compound had H-bonds with the residues GLY143 with length 1.59Å, CYS145 with length 1.59Å, GLU166 with length 1.47Å and a bond with an aromatic ring with length 1.69Å with the residue HIE41.

Dolutegravir presented H-bonds with the residues GLU166 with a length of 1.48Å. Other H-bonds were with the residues GLY143 with a length of 1.55Å and GLN189 with a length of 1.73Å (see Table 1).

**Table 1.** Docking outcomes of H-bonds, bond lengths and docking score for the compound selected.

Compound	H-bonds	Bond lengths	Docking score
Zidovudine	CYS145, GLU166	1.64Å, 1.72Å	-6.241
Tromantadine	LEU141, GLN189, GLU166	1.62Å, 1.54Å, 1.59Å	-6.229
Cobicistat	GLU166, GLY143, HIE41	1.61Å, 1.53Å, 1.71Å	-8.837
Doravirine (Pifeltro)	CYS145, GLU166, HIE41, GLY143	1.59Å, 1.47Å, 1.69Å, 1.59Å	-7.471
Dolutegravir	GLU166, GLY143, GLN189	1.48Å, 1.55Å, 1.73Å	-7.152
Indinavir	GLU166, HIE41	1.43Å, 1.53Å, 1.68Å	-6.618

Finally, Figure 7 shows the docking outcomes for the indinavir ligand. This compound had two interactions with the residue GLU166 with lengths of 1.43Å and 1.53Å. On the other hand, this ligand had a H-bond with the residue HIE41 with a length of 1.68Å.

### Electrostatic potential comparison

**Figure 6.**
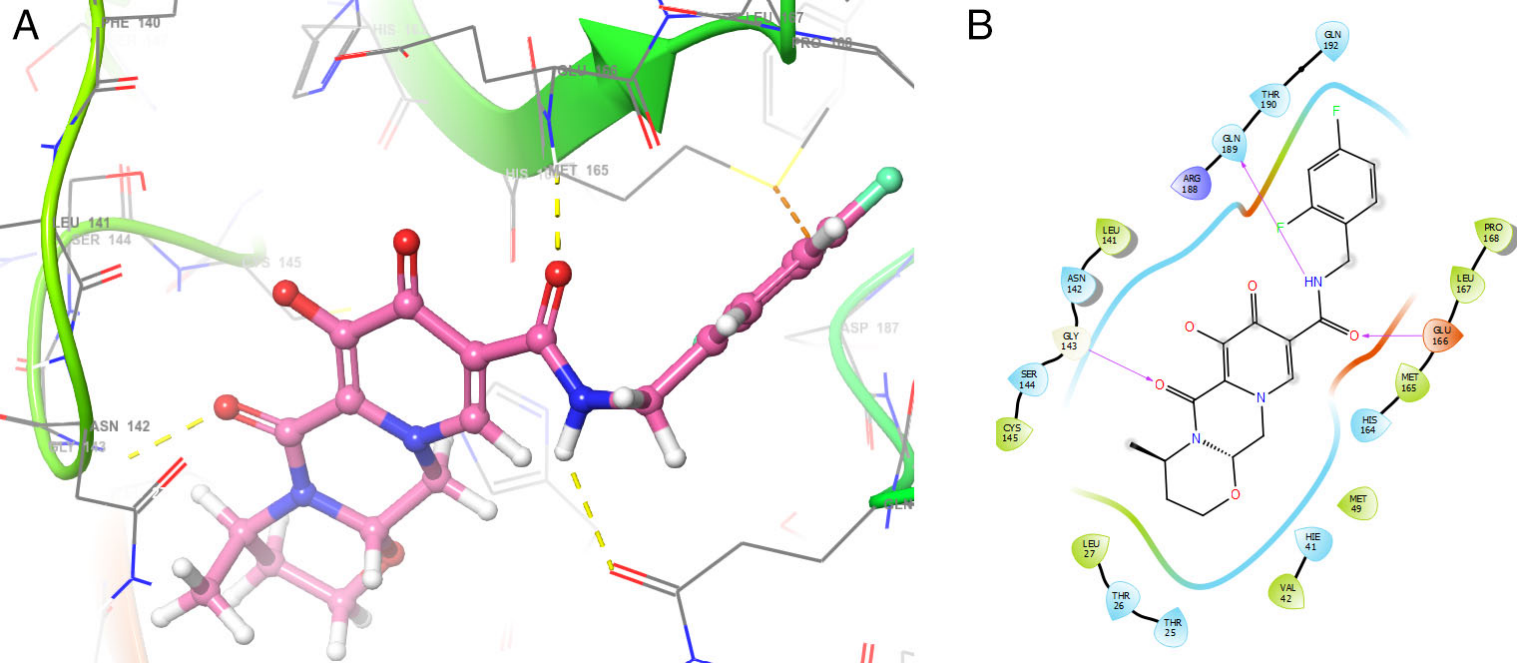
Molecular docking outcomes (A) and interaction (B) for dolutegravir.

In the previous section, we have seen that the interactions that form between the ligands and the RNA polymerase can be classified into two main types, purely electrostatic attractions and interactions by delocalisation of charges. In this and the following section, we develop these aspects using DFT calculations. Figure 8 shows the function ESP for the ligands zidavudine, cobicistat and dolutegravir. In the case of zidavudine the ESP on the O2 and O3 atoms has a clear correspondence with the interactions with CYS145 and GLU166 which can be seen in Figure 2. In the case of cobicistat the significant values of the ESP function on the O4 and O7 atoms have a correspondence with the interactions with GLU166 and GLY143 ( Figure 4) respectively. Finally, in the case of dolutegravir, the ESP function calculated on the O4 and O7 atoms had a clear agreement with the interactions with CYS145 and GLU166 respectively, which can be seen in Figure 6.

**Figure 7.**
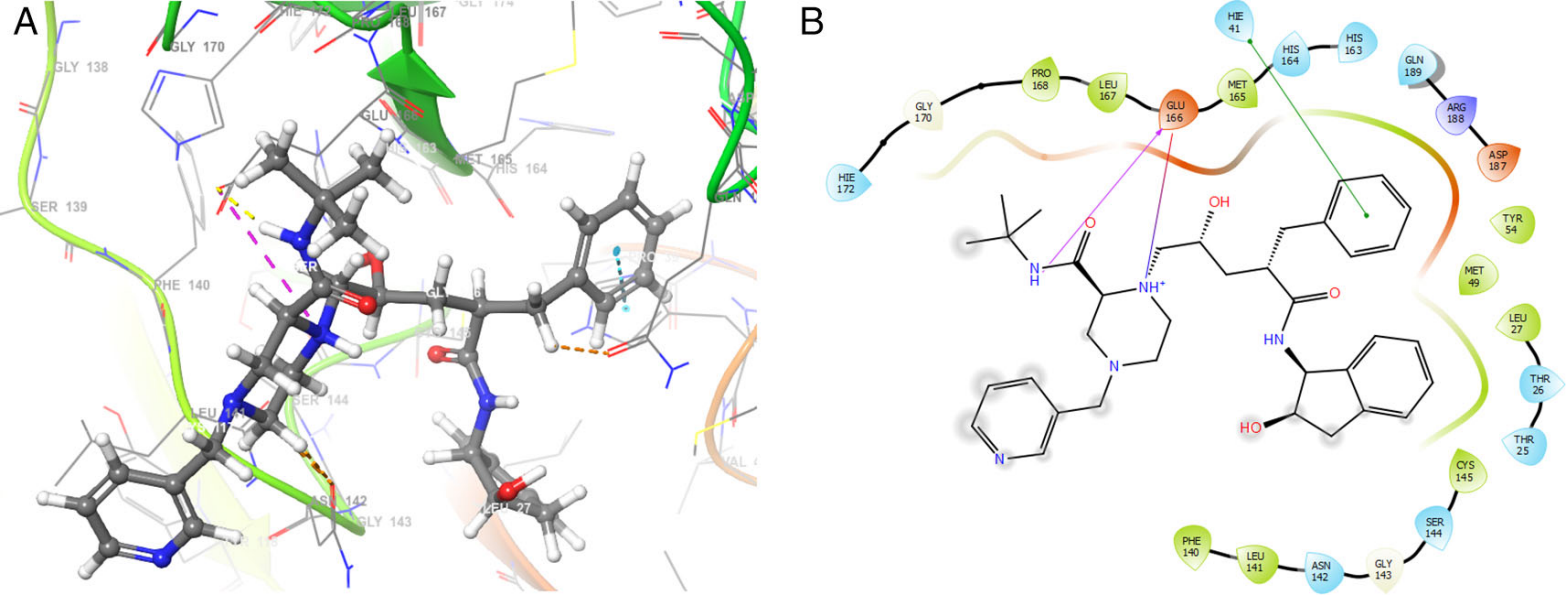
Molecular docking outcomes (A) and interaction (B) for indinavir.

Figure 9 shows the Function ESP for the ligands tromantadine, doravirine and indavir. In the case of tromantadine, the ESP on the O1 and N4 atoms have a clear correspondence with the interactions with GLU166 and LEU141 which can be seen in Figure 3. In the case of doravirine the significant values of the ESP function on the O6 and N10 atoms had a correspondence with the interactions with GLU166 and GLY143 ( Figure 5) respectively. Finally, in the case of indavir, the ESP function calculated on the N5 atom had a clear correspondence with the interaction with GLU166, which can be seen in Figure 7. In Figures S13-S24 ( *Extended data* ^43^) 10.6084/m9.figshare.22670167.v1, the images of all the ESPs for all the ligands studied can be seen.

**Figure 8.**
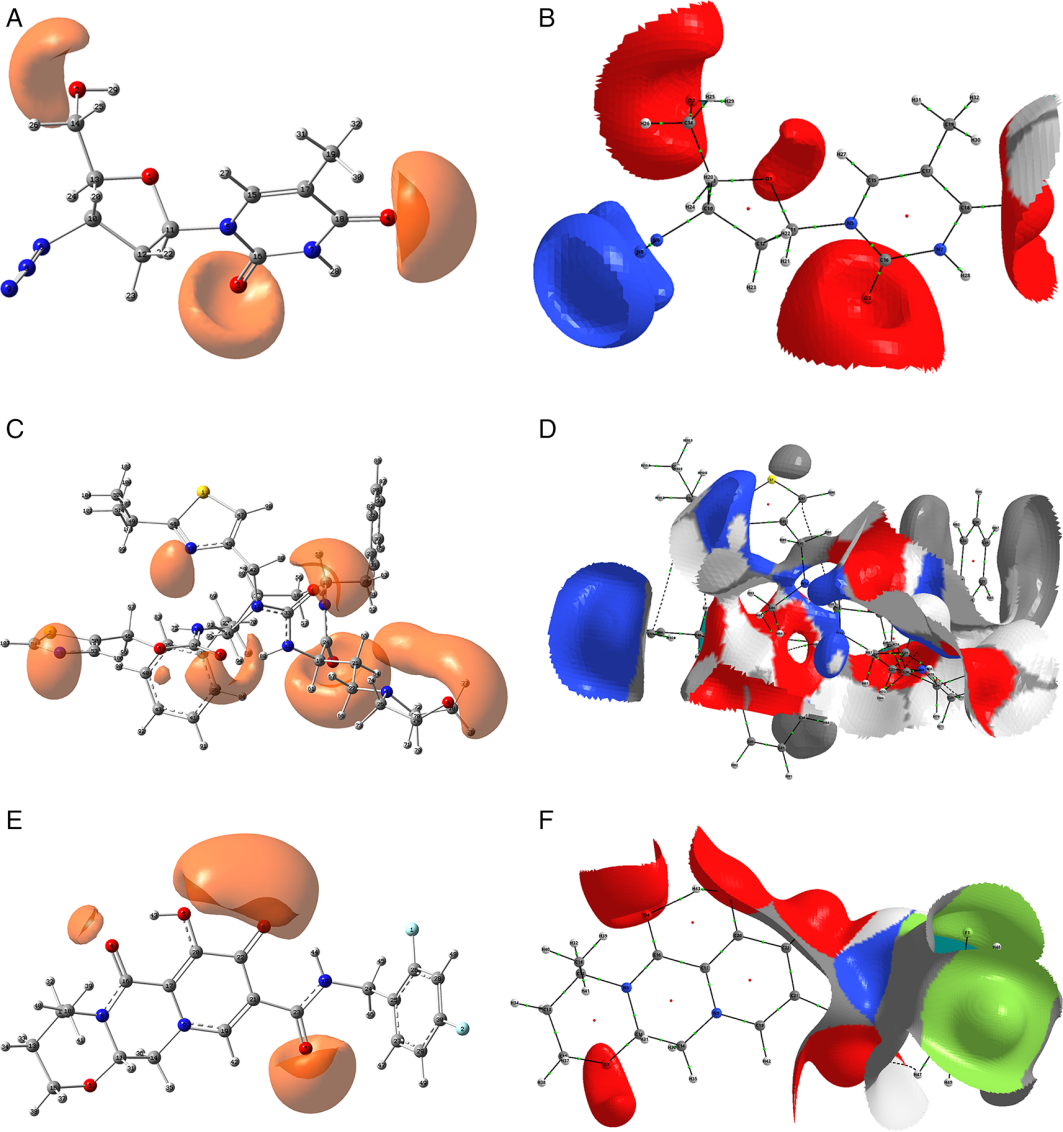
Function ESP for the ligands: A) Zidavudine, B) Zidavudine but with the different contributions of each atom to the ESP represented by colours, C) Cobicistat, D) The same as B but for cobicistat, E) Dolutegravir and F) The same as B but for dolutegravir. The isovalue for A, C and D was -0.04, and the iso. for B, D and E was -0.01. Figures A, C and D were created using GaussView 5.0 and B, D and E using AIMAll (v. 17.11.14).

**Figure 9.**
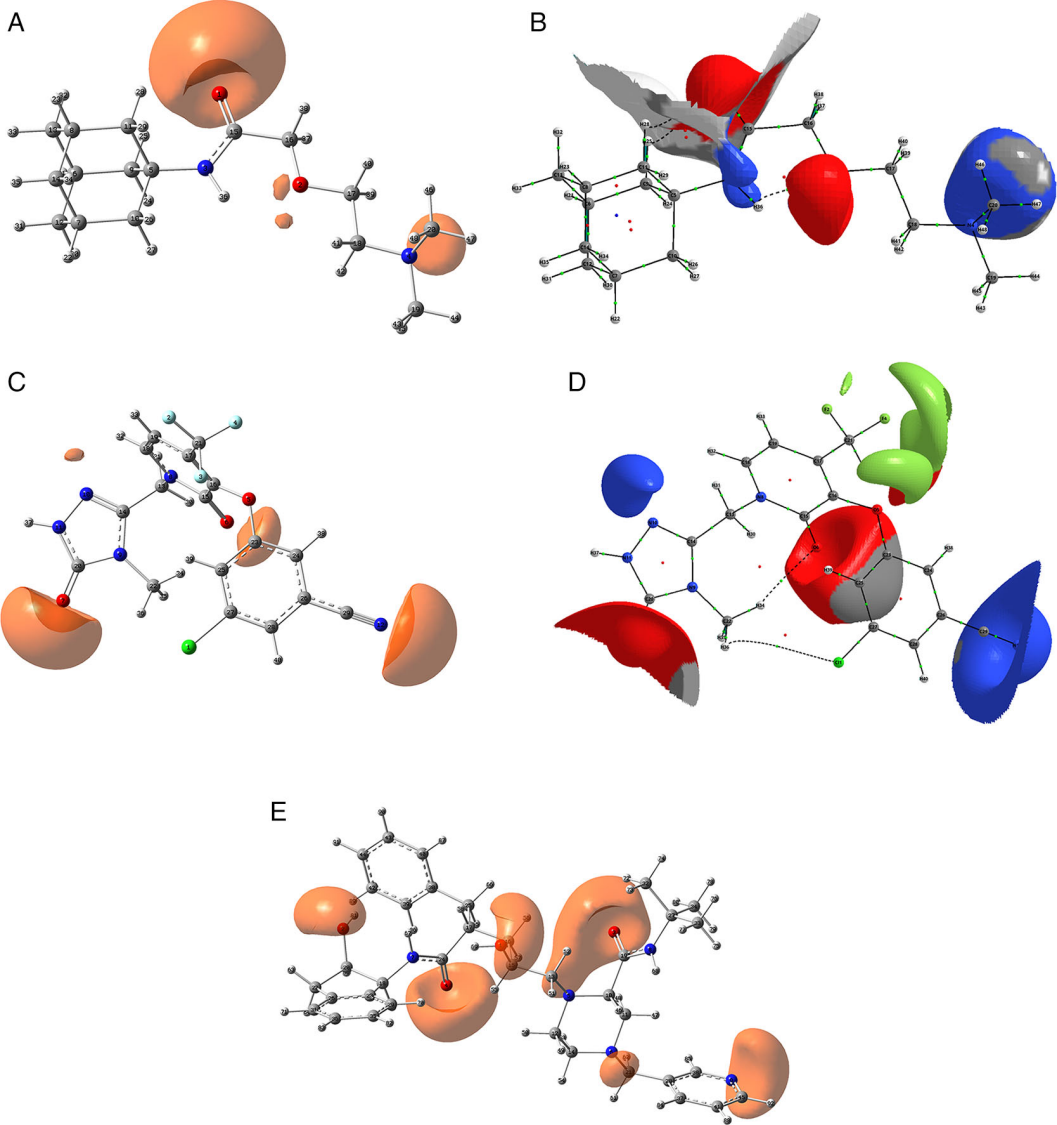
Function ESP for the ligands: A) Tromantadine B) Tromantadine but with the different contributions of each atom to the ESP represented by colours, C) Doravirine, D) The same as the B but for doravirine, E) Indavir and F) The same as the B but for indavir. The isovalue for A, C and D was -0.04, and the iso. for B, D and E was -0.01. Figures A, C and D were created using GaussView 5.0 and B, D and E using AIMAll (v. 17.11.14).

### Global reactivity descriptors analysis and Fukui function comparison

The study also investigated the global and local chemical reactivity descriptors using DFT calculations. Table 2 presents the calculated global parameters, including chemical potential, chemical hardness, global softness, and global electrophilicity, to compare the chemical reactivity of the ligand sample. As indicated in Table 2, the least reactive molecule is tromantidine, exhibiting the lowest values of electronic chemical potential *μ*, softness *S*, and electrophilicity *ω* (for the chemical potential it refers to its absolute value). On the other hand, it has the highest chemical hardness ( *η*) value. The most reactive compounds are pyramidine and doravirine with the highest values for electronic chemical potential, softness and electrophilicity, as well as the lowest chemical hardness values. The electrophilicity values can have a crucial influence on the stability of the active site of ligands that are stabilized by non-covalent interactions.

**Table 2.** Global reactivity descriptors (in eV) for the sample of ligands.

Compound	Chemical potential ( *μ*), eV	Chemical hardness *(η*), eV	Softness ( *S*), eV	Electrophilicity ( *ω*), eV
Amprenavir	-2.9264	4.1233	0.2425	1.0385
Boceprevir	-3.3438	3.8677	0.2586	1.4454
Cobicistat	-2.7986	3.3281	0.3005	1.1767
Dolutegravir	-3.4005	3.2621	0.3066	1.7724
Doravirine	-3.7872	3.2243	0.3101	2.2241
Hydroxychoroquine	-3.0790	3.3595	0.2977	1.4110
Indinavir	-2.8490	3.9519	0.2530	1.0270
Oseltamivir	-3.1891	4.1103	0.2433	1.2372
Pyramidine	-3.7903	2.4376	0.4102	2.9468
Tromantadine	-2.6287	4.4582	0.2243	0.7750
Zalcitabine	-2.8476	4.1341	0.2419	0.9807
Zidovudine	-3.4311	4.1407	0.2415	1.4215
Truvada	-3.2542	4.3598	0.2293	1.1248
Trizivir	-2.5847	4.2689	0.2342	1.1582
Trifluridine	-2.8754	4.4254	0.2259	1.1458
Sofosbuvir	-3.4875	4.3564	0.2295	1.1368

**Figure 10.**
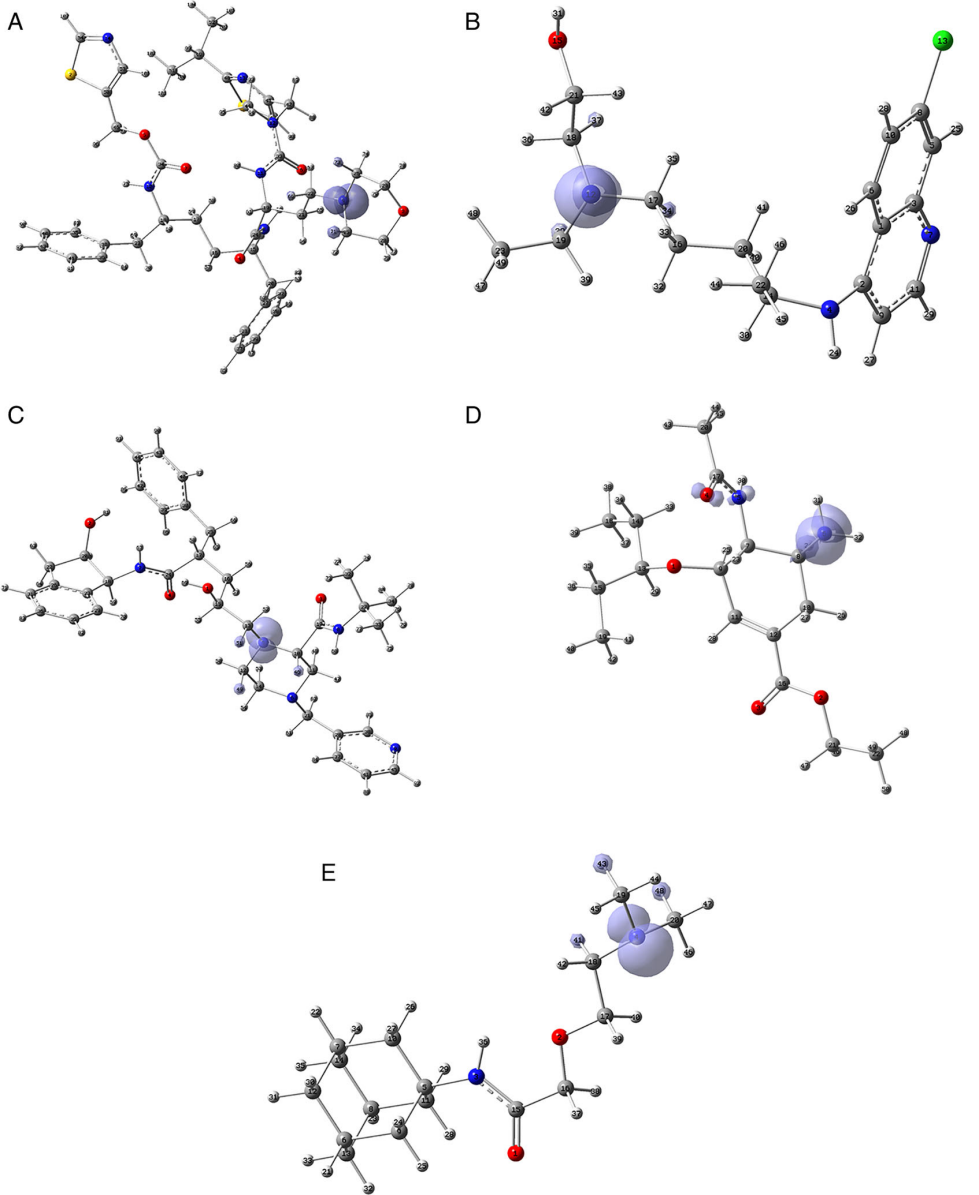
Fukui function $f^{-}(\vec{r})$ calculated under the FMO approximation $\left({\left|\mathrm{HOMO}(\vec{r})\right|}^{2}\right)$ for the ligands. A) Cobicistat, B) Hydroxychoroquine, C) Indinavir, D) Oseltamivir and E) Tromantadine. Isovalue was 0.01 in all cases. The figure was created using GaussView 5.0.

Since the analysis of the global parameters is limited, we will complete it with the comparison of some local descriptor functions. The electrophile and nucleophile Fukui functions (as a measure of reactivity) were then compared using the Frontier Molecular Orbital (FMO) approach. The electrophilic-nucleophilic character of the following functions also shows those molecular areas that are most likely to form charge-donating interactions (basically by charge delocalisation). These types of interactions are important and difficult to determine using docking analysis. Figure 10 shows the functions for the compounds cobicistat, hydroxychoroquine, indinavir, oseltamivir and tromantadine (A-E respectively), it can be noted that in these five cases the function assigns the most nucleophilic character to a nitrogen atom, mainly to its unshared electron pair. When comparing this figure with Figures 3 and 7 we can see that some important interactions can be justified on this basis; for example, in the case of Indinavir the N5 has an important interaction with GLU166, or in the case of Tromantadine the N4 has an important interaction with LEU141.

Figure 11 shows that in the case of zidavudine the O3 would have an important interaction as a charge donor with GLU166 ( Figure 2). For doravirine, the N10 has an interaction with GLY143 and the N11 with CYS145 ( Figure 5). In the case of dolutegravir we have not found any match for the function $f^{-}\left(\vec{r}\right)$ . In Figures S25-S36 ( *Extended data* ^43^), we can see images of all the functions $f^{-}\left(\vec{r}\right)$ for all the ligands studied.

**Figure 11.**
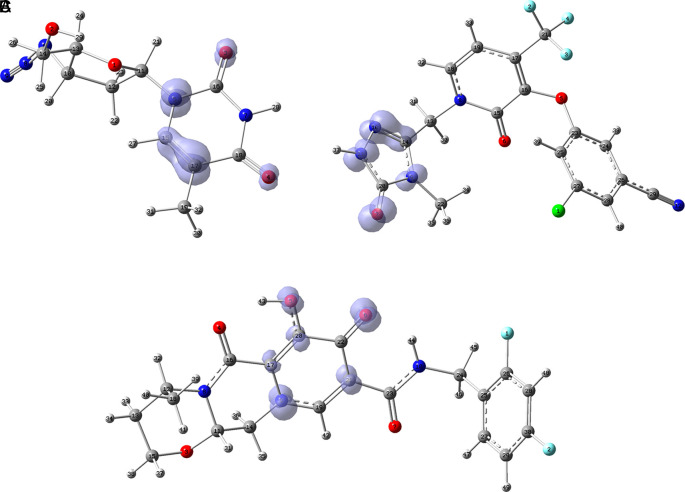
Fukui function $f^{-}(\vec{r})$ calculated under the FMO approximation $\left({\left|\mathrm{HOMO}(\vec{r})\right|}^{2}\right)$ for the ligands. A) Zidavudine, B) Doravirine and C) Dolutegravir. The isovalue was 0.01 in all cases. The figure was created using GaussView 5.0.

**Figure 12.**
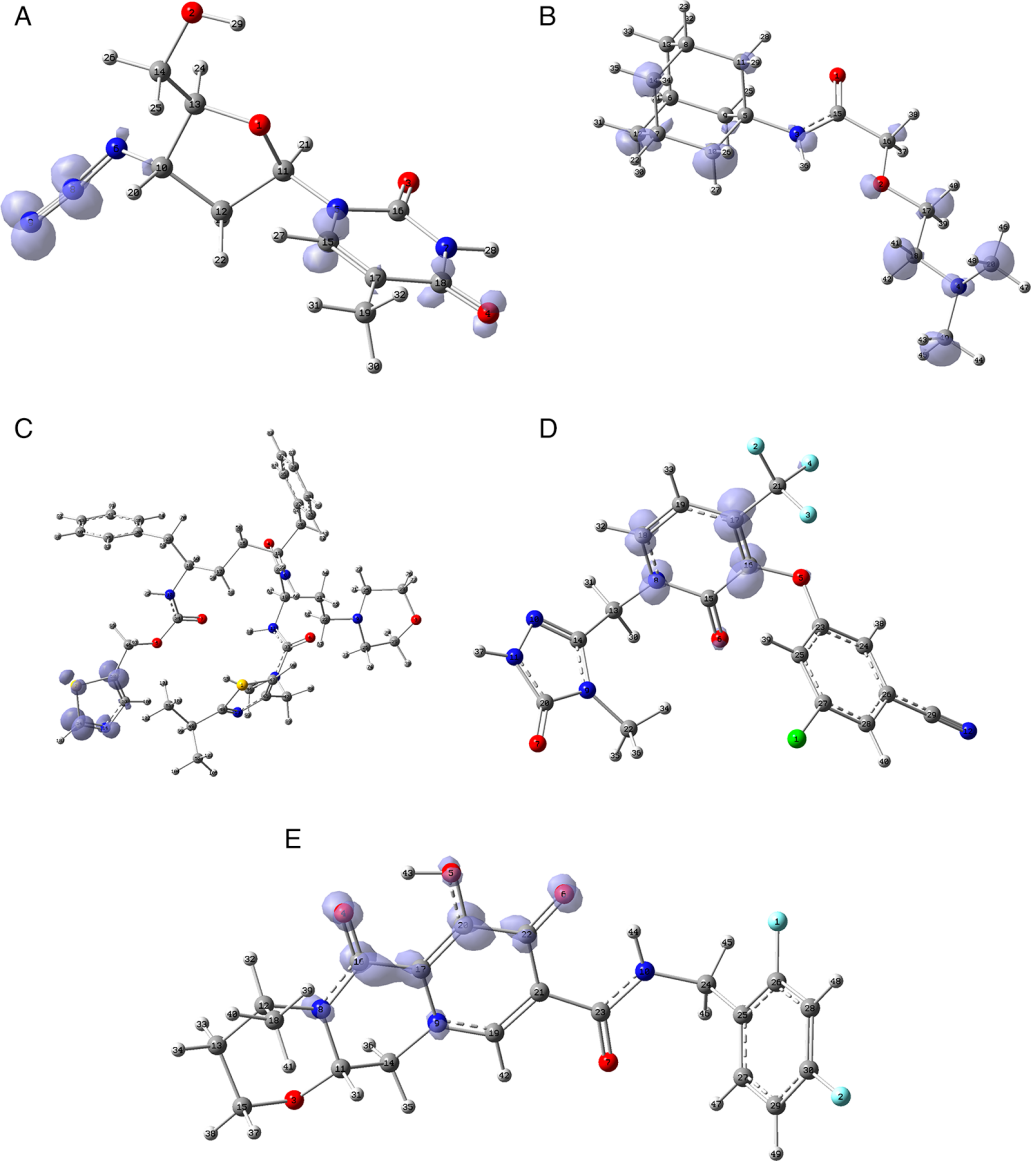
Fukui function $f^{+}(\vec{r})$ calculated under the FMO approximation $\left({\left|\mathrm{LUMO}(\vec{r})\right|}^{2}\right)$ for the ligands. A) Zidavudine, B) Tromantadine, C) Cobicistat, D) Doravirine and E) Dolutegravir. The isovalue was 0.01 in all cases. The figure was created using GaussView 5.0.

Figure 12 shows the functions $f^{+}\left(\vec{r}\right)$ calculated under the FMO approximation $\left({\left|\text{LUMO}\left(\vec{r}\right)\right|}^{2}\right)$ for compounds **A**) Zidavudine, **B**) Tromantadine, **C**) Cobicistat, **D**) Doravirine, **E**) Dolutegravir and **F**) Indavir. In the case of tromantadine, the Fukui function $f^{+}\left(\vec{r}\right)$ on the N3 and N4 atoms justifies interactions by charge attraction towards these atoms with GLN189 and LEU141 respectively. In the case of dolutegravir, the value of the function on O4 indicates a possible interaction by charge delocalisation with GLU143. For the rest of the ligands no matches for the function $f^{+}\left(\vec{r}\right)$ were found. In Figures S25-S36 in the supporting information ( *Extended data* ^43^) are images of all the functions $f^{+}\left(\vec{r}\right)$ of all the ligands studied.

## Conclusions

The present investigation involved the analysis of a set of compounds (Zidovudine, Tromantadine, Pyramidine, Oseltamivir, Hydroxychoroquine, Cobicistat, Doravirine, Dolutegravir, Boceprevir, Indinavir, Truvada, Trizivir, Trifluridine, Sofosbuvir and Zalcitabine) employed in *in vitro* studies against SARS-CoV-2. Molecular docking, comparison of electrostatic potentials, and evaluation of chemical reactivity functions were conducted to examine the active site stabilization interactions of these compounds from both structural and electronic perspectives.

From the molecular docking results, it was observed that tromantadine, dolutegravir, cobicistat, doravirine and dolutegravir show good active site stabilization with at least one H-bond in each conformation. To further investigate the active site stabilization of each ligand, a DFT reactivity analysis and electrostatic potential comparison was developed.

By utilizing the crystal structure of SARS-CoV-2 RNA-dependent RNA polymerase, these analyses enabled the identification of the primary stabilizing interactions. This research presents novel insights into these ligands, which can be advantageous in the development of new treatments for COVID-19. The studies allowed us to find an explanation supported in the DFT about the chemical reactivity and the stabilization in the active site of the ligands. The interactions between the ligands and the RNA polymerase studied were of two main types: electrostatic (usually hydrogen bonding) and charge delocalisation interactions. Both types of interactions coexist in these superstructures and form strong interactions that adequately justify the inhibitory activity of these ligands.

## Data Availability

Harvard Dataverse: Data for Receptor-Based Pharmacophore Modelling of a series of ligands used as inhibitors of the SARS-CoV-2 virus by complementary theoretical approaches, molecular docking, and reactivity descriptors, https://doi.org/10.7910/DVN/IA8EOB. ^42^ Data are available under the terms of the Creative Commons Zero “No rights reserved” data waiver (CC0 1.0 Public domain dedication). Figshare: Supporting Information.docx, https://doi.org/10.6084/m9.figshare.22670167.v1. ^43^ Data are available under the terms of the Creative Commons Attribution 4.0 International license (CC-BY 4.0).
