# Supplementary material for: Receptor-Based Pharmacophore Modelling of a series of ligands used as inhibitors of the SARS-CoV-2 virus by complementary theoretical approaches, molecular docking, and reactivity descriptors
Source: F1000Res. 2023 Jun 26;12:749. [Version 1] doi: 10.12688/f1000research.133426.1 (PMC11406136; doi:10.12688/f1000research.133426.1)
Supplement: Peer review history

# Reviewer report: Reviewer response for version 1

**DOI:** 10.5256/f1000research.146415.r216363

**Published:** 2024-09-16

**Authors:** Shiva Prasad Kollur

**Affiliations:** 1 Amrita Vishwa Vidyapeetham, Coimbatore, Tamil Nadu, India

Alejandra and Jesus in this manuscript titled "Receptor-Based Pharmacophore Modelling of a series of ligands used as inhibitors of the SARS-CoV-2 virus by complementary theoretical approaches, molecular docking, and reactivity descriptors" have identified the stabilizing interactions using the crystal structure of SARS‑CoV‑2 RNA‑dependent RNA polymerase. Based on the results obtained, the ligands studied in the present work can be used in the design of new COVID-19 treatments. Furthermore, the studies are also supported by the Density Functional Theory about the chemical reactivity and the stabilization in the active site of the ligands.

 This work is highly significant and authors have written the same in a scientifically well-manner.

 I recommend the approval of this manuscript in the current form.

Is the work clearly and accurately presented and does it cite the current literature?

Yes

If applicable, is the statistical analysis and its interpretation appropriate?

No

Are all the source data underlying the results available to ensure full reproducibility?

Yes

Is the study design appropriate and is the work technically sound?

Yes

Are the conclusions drawn adequately supported by the results?

Yes

Are sufficient details of methods and analysis provided to allow replication by others?

Yes

Reviewer Expertise:

Chemical biology, chemical synthesis, computational chemistry and materials chemistry.

I confirm that I have read this submission and believe that I have an appropriate level of expertise to confirm that it is of an acceptable scientific standard.

# Reviewer report: Reviewer response for version 1

**DOI:** 10.5256/f1000research.146415.r216355

**Published:** 2023-11-13

**Authors:** Syeda Rehana Zia

**Affiliations:** 1 University of Karachi, Karachi, Pakistan

Comments:

 The manuscript holds merit in the realm of SARS-CoV-2 ligand identification. However, the authors should encounter the following comments for further improvement and potential recommendation of the manuscript:

1. The authors should enhance the introduction by explicitly stating the significance of their research. Additionally, providing a concise overview of the research conducted would strengthen the connection between the literature review, past studies, and the current investigation. This adjustment would contribute to a clearer understanding of the study's background and purpose.
2. The authors have not mentioned details about pharmacophoric generation in the method section. However, a detailed explanation is required for clarity and reproducibility.
3. The authors have mentioned in the result section about the pharmacophore development of Zidovudine, Tromantadine, Pyramidine, Oseltamivir, Hydroxychoroquine, Cobicistat, Doravirine (Pifeltro), Dolutegravir, Boceprevir, Indinavir, Truvada, Trizivir, Trifluridine, Sofosbuvir and Zalcitabine. However, in Figure 1 pharmacophore development for only four ligands have been demonstrated namely Truvada, Trizivir, Trifluridine and Sofosbuvir. The authors should provide a rationale for displaying only four ligand pharmacophores. Clarifying the selection criteria or limitations would enhance the readers insight.
4. In the result section, it is written “The validation process yielded an RMSD value of 0.38”; however, it's not clear whether your RMSD value of 0.38 is from a pharmacophore modeling or molecular docking study. RMSD can be used to measure the similarity either between the pharmacophoric features of a reference (known) ligand and a predicted pharmacophore model or between the predicted binding pose of a ligand compared to its experimentally determined pose. Secondly, the unit for RMSD and docking score is also missing in the text.
5. The docking and ESP outcomes for ligands: Truvada, Trizivir, Trifluridine and Sofosbuvir, with developed pharmacophores, are not reported in the manuscript either in main text or in supplementary data. This omission might limit the comprehensive assessment of pharmacophore model’s predictive power. In other case if authors have used these ligands as a "training set", they should mention it in the manuscript.
6. The authors have mentioned in the supplementary data about molecular docking and ESP calculation for Amprenavir, however, its rationale is not mentioned in the text. The authors should state why they have performed these calculations and how it is related to their study.
7. The authors have written that: “The best predictions of the poses were predicted by 10ns molecular dymane calculations, in order to analyse its stabilization in the active site.” However, absence of details regarding MD simulations in methods and results is noted.
8. There are certain grammatical mistakes in the manuscript, for instance “de” should be replaced with “the” etc.

Is the work clearly and accurately presented and does it cite the current literature?

Partly

If applicable, is the statistical analysis and its interpretation appropriate?

Not applicable

Are all the source data underlying the results available to ensure full reproducibility?

Partly

Is the study design appropriate and is the work technically sound?

Yes

Are the conclusions drawn adequately supported by the results?

Yes

Are sufficient details of methods and analysis provided to allow replication by others?

Partly

Reviewer Expertise:

Computational Chemistry,

I confirm that I have read this submission and believe that I have an appropriate level of expertise to confirm that it is of an acceptable scientific standard, however I have significant reservations, as outlined above.

# Reviewer report: Reviewer response for version 1

**DOI:** 10.5256/f1000research.146415.r216353

**Published:** 2023-10-27

**Authors:** Mohammad Mahfuz Ali Khan Shawan

**Affiliations:** 1 Jahangirnagar University, Savar, Dhaka Division, Bangladesh

The manuscript titled 'Receptor-Based Pharmacophore Modelling of a series of ligands used as inhibitors of the SARS-CoV-2 virus by complementary theoretical approaches, molecular docking, and reactivity descriptors' is engaging and well-structured. It tackles a current issue by proposing a therapeutic solution. The authors employ diverse bioinformatic tools and techniques to repurpose various antiviral agents against the RdRp of SARS-CoV-2. The overall findings are promising and hold potential for effective COVID-19 treatments. The authors' commitment to addressing this crucial disease is commendable. However, certain aspects of the manuscript require further clarification. Notable concerns are outlined below and must be resolved prior to acceptance.

1. The current title of the manuscript is so hard to get.  It's suggested to make the title clearer. The proposed new title is: “In Silico Receptor-Based Pharmacophore Modelling against COVID-19 infection: targeting the RdRp of SARS-CoV-2”. This new title highlights and focuses on the repurposing of antiviral drugs and targeting the RdRp of SARS-CoV-2 to combat COVID-19 infection using computational methods.
2. Revise and improve the abstract to enhance its clarity and completeness. Integrate a brief description underscoring the significance of selecting COVID-19's RdRp as a focal point. Conclude the abstract by emphasizing the pressing need for hands-on laboratory investigations to validate the outcomes presented in this manuscript. A few words that summarize the importance of this study and future scope should also be incorporated within the abstract section as concluding remarks.  
3. In the introduction section, there is an error on page 3, line 11. Please remove “For further information, refer to the question-and-answer section on dexamethasone”. Additionally, it is important for the authors to review and reference recent studies that focus on developing effective components against SARS-CoV-2, particularly targeting the RdRp. By doing so, the current study can strengthen its significance and relevance by acknowledging existing findings and gaps. The introduction section should be more detailed and focused on the materials and methods that have been adopted in this study and adjusted to align with the revised abstract, maintaining consistency between the two. Furthermore, the importance of computational studies should be added.
4. In the "Materials and Methods" section, it's recommended to include a flow chart for an early bird overview of the computational approach. This chart should detail the step-by-step process, including the software, server names, and addresses used for in-silico identification of potent anti-COVID-19 drugs. To facilitate replication, authors should provide specifications of the computational workstation they employed. For accurate molecular docking, proper preparation of the active site and grid box is necessary, along with validation of the docking method. After molecular docking, molecular dynamics must need to be included to support the findings. To bolster the results, authors should incorporate studies on the molecular targets and biological activity analysis of the tested antivirals. They should also consider including established anti-COVID-19 antiviral drugs (such as paxlovid, molnupiravir, remdesivir, and camostat mesylate) as controls, and compare their outcomes against these controls. Furthermore, the materials and methods section needs caution and extensive rewriting, there are many missing details and several mistakes too. The authors can find the following relevant articles to enhance their "Materials and Methods" section and incorporate citations to support their revised manuscript.  
https://doi.org/10.1186/s40064-016-2996-5 (May be used for active site analysis using CastP web server).
https://doi:10.1016/j.meegid.2021.104951 (May be used for molecular dynamics)
https://doi.org/10.1186/s42269-020-00479-6 (May be used for molecular dynamics)
https://doi.org/10.1155/2023/5469258 (May be used for molecular dynamics)
https://doi.org/10.1007/s10989-023-10535-0 (May be used for molecular dynamics)
5. In the results section, there is a need to enhance the quality of the figures, particularly focusing on different atom's names and positions. The figures with text display blurry fonts, thus necessitating their re-exportation at a higher resolution. These improved figures should then be integrated into the revised article to ensure clarity for readers.
6. The conclusion section contains scientific inaccuracies and could benefit from additional clarity. Consider enhancing its comprehensibility. It might be beneficial to introduce a discussion section as well, addressing the potential for future improvements and strategies to enhance effectiveness in upcoming endeavors.
7. It is recommended to thoroughly review all references in the manuscript for typographical errors. Furthermore, ensure that the reference section adheres to the formatting guidelines specified by the journal.
8. The manuscript exhibits a lack of proficiency in English language usage encompassing grammar and writing skills. It is imperative to engage an expert language editing service or a native speaker adept in writing to thoroughly review the entire manuscript. A comprehensive revision is essential to enhance the manuscript's overall command of the English language.
9. Please submit the revised manuscript after conducting a plagiarism check that yields a result between 10% and 15%.

Is the work clearly and accurately presented and does it cite the current literature?

Partly

If applicable, is the statistical analysis and its interpretation appropriate?

Not applicable

Are all the source data underlying the results available to ensure full reproducibility?

Partly

Is the study design appropriate and is the work technically sound?

Yes

Are the conclusions drawn adequately supported by the results?

Yes

Are sufficient details of methods and analysis provided to allow replication by others?

Partly

Reviewer Expertise:

Bioinformatics

I confirm that I have read this submission and believe that I have an appropriate level of expertise to confirm that it is of an acceptable scientific standard, however I have significant reservations, as outlined above.

## References

[1] : Halogen-directed drug design for Alzheimer's disease: a combined density functional and molecular docking study. *Springerplus* .2016;5(1) : 10.1186/s40064-016-2996-5 1346 10.1186/s40064-016-2996-5 27588239 PMC4987752

[2] : Designing an effective therapeutic siRNA to silence RdRp gene of SARS-CoV-2. *Infect Genet Evol* .2021;93: 10.1016/j.meegid.2021.104951 104951 10.1016/j.meegid.2021.104951 34089909 PMC8170914

[3] : Luteolin and abyssinone II as potential inhibitors of SARS-CoV-2: an in silico molecular modeling approach in battling the COVID-19 outbreak. *Bull Natl Res Cent* .2021;45(1) : 10.1186/s42269-020-00479-6 27 10.1186/s42269-020-00479-6 33495684 PMC7816153

[4] : In Silico Identification and Analysis of Potentially Bioactive Antiviral Phytochemicals against SARS-CoV-2: A Molecular Docking and Dynamics Simulation Approach. *Biomed Res Int* .2023;2023: 10.1155/2023/5469258 5469258 10.1155/2023/5469258 37214084 PMC10195178

[5] : Advances in Computational and Bioinformatics Tools and Databases for Designing and Developing a Multi-Epitope-Based Peptide Vaccine. *Int J Pept Res Ther* .2023;29(4) : 10.1007/s10989-023-10535-0 60 10.1007/s10989-023-10535-0 37251529 PMC10203685
